# First-Principles Density Functional Theory Study of Modified Germanene-Based Electrode Materials

**DOI:** 10.3390/ma15010103

**Published:** 2021-12-23

**Authors:** Xue Si, Weihan She, Qiang Xu, Guangmin Yang, Zhuo Li, Siqi Wang, Jingfei Luan

**Affiliations:** 1 School of Physics, Changchun Normal University, Changchun 130032, China; sx1657663198@163.com (X.S.); s17543055883@163.com (W.S.); lz1327499805@163.com (Z.L.); w348643062@163.com (S.W.); 2 School of Prospecting and Surveying, Changchun Institute of Technology, Changchun 130021, China; xuqiang19810919@163.com; 3 State Key Laboratory of Pollution Control and Resource Reuse, School of the Environment, Nanjing University, Nanjing 210008, China

**Keywords:** germanene, supercapacitors, adsorption, quantum capacitance

## Abstract

Germanene, with a wrinkled atomic layer structure and high specific surface area, showed high potential as an electrode material for supercapacitors. According to the first-principles calculation based on Density Functional Theory, the quantum capacitance of germanene could be significantly improved by introducing doping/co-doping, vacancy defects and multilayered structures. The quantum capacitance obtained enhancement as a result of the generation of localized states near the Dirac point and/or the movement of the Fermi level induced by doping and/or defects. In addition, it was found that the quantum capacitance enhanced monotonically with the increase of the defect concentration.

## 1. Introduction

With the progress of science and technology in modern society, energy shortage is becoming increasingly serious. For example, some fossil fuels are already in short supply. Therefore, the search for recyclable and regenerable energy sources is imminently needed. The development of high-performance electrical energy storage devices, such as rechargeable batteries and electrochemical capacitors [1,2], has become a research hotspot. The electrochemical double-layer capacitors (EDLCs) [3,4,5,6,7,8,9,10] have the advantages of a simple charging circuit, long life, and high output power [11], but low energy density. Thus, the efficient supercapacitors should have an electrochemically stable electrode-electrolyte interface with high specific surface/interface area to enhance the energy density. Researchers are constantly trying to explore new electrode materials or modify existing materials to improve the performance of supercapacitors [12].

Graphene is a typical two-dimensional electrode material [13,14]. However, graphene exhibits certain drawbacks in the application process, such as poor accessibility to the electrolyte [13,15]. Hence, it is particularly important to find new two-dimensional materials.

Doping and functionalization are the usual methods to improve the capacitance of graphene [16]. As two-dimensional layered nanosheet materials, silicene and germanene have attracted attention due to the inspiration of graphene. As far as spin-orbit interaction (SOI) was concerned, germanene was more prominent than silicene and graphene, the SOI of germanene, silicene and graphene were 46.3 meV, 4 meV and 1 μeV, respectively [17,18,19]. The large spin-orbit gap (24 meV) of germanene made it a typical alternative material for exhibiting the Quantum Spin Hall Effect [20,21,22]. Furthermore, germanene was easier to be functionalized and had been synthesized by different chemical methods [23,24,25,26,27,28]. Thus, germanene had become a major competitor in the EDLCs electrode materials.

Recent experimental and theoretical results had shown that the total interface capacitance (*C_T_*) in supercapacitors is affected by both the quantum capacitance (*C_Q_*) and electric double layer (*C_D_*) capacitance [29,30,31]. The increase of quantum capacitance is an effective method for improving the total interface capacitance. However, the *C_Q_* of germanene-based electrodes materials for supercapacitors has not been fully studied. In the application process and/or growth conditions, the introduction of vacancy defects [32,33,34], doping/co-doping [35,36,37,38,39,40,41], and adsorbents [42,43] could alter the electronic structure, thereby affecting the quantum capacitance. Furthermore, the randomness of defects/doping had an influence on the electronic and transport properties, for example, edge defects have an effect on the transport gap [44,45]. We will expand this content in the next study. In the previous work, we studied different types of vacancy defects and metal atoms adsorbed on single-vacancy germanene. However, there were few studies on the adsorption of pristine germanene, concentration effects and co-doping. Therefore, this paper explored *C_Q_* of pristine or defected germanene with monolayer/multilayer structures on the basis of Density Functional Theory. At the same time, the influence of defect concentration on the quantum capacitance was discussed. The calculation results indicated that both vacancies and doping/co-doping could improve the quantum capacitance. According to these results, we analyzed the reasons for the performance enhancement of electrode materials with different defect structures.

## 2. Materials and Methods

All the calculations were performed by the PAW (Projector Augmented Wave) potential [46] method on the basis of DFT as implemented in the VASP code [47]. The generalized gradient approximation with the parametrization of Perdew–Burke–Ernzerhof (PBE) [48] is used to express the electron exchange correlation energy of the interaction [49]. The calculation of the PBE generalized function was found to be reliable by Zhu et al. [50]. To ensure that the total energy converges to 1 meV/atom, the k-space integral and plane-wave basis were chosen [51]. It was found that the kinetic energy cut-off value of plane wave expansion was 450 eV, which had significant influence. The Monkhorst–Pack method was used to sample K-points in the Brillouin zone [49]. The spin was also considered in the calculation process.

Through sp^3^/sp^2^-hybridization, the lattice constant of the buckled honeycomb structure of pristine germanene was 4.061 Å. According to our calculations, the Ge-Ge bond length was found to be 2.443 Å, which was similar to the previous studies [52,53]. Based on the pristine cell containing two atoms, the article established different supercells as ideal models, including 2 × 2 (containing 8 atoms), 3 × 3 (containing 18 atoms), 4 × 4 (containing 32 atoms), 5 × 5 (containing 50 atoms) and 6 × 6 (containing 72 atoms) hexagonal structures. We calculated the effects of metallic atoms Ti, Au, Ag, Cu, Al and non-metallic atoms B, N, P, S on quantum capacitance.

To avoid layer-to-layer interaction, we chose a vacuum space of 18 Å in the supercell. The Brillouin zones of 2 × 2, 3 × 3, 4 × 4, 5 × 5 and 6 × 6 supercells were sampled with a Γ-centered k-point grid of 24 × 24, 16 × 16, 12 × 12, 10 × 10, 6 × 6, respectively.

The *C_Q_* can be expressed as *C_Q_* = *dσ*/*d*Φ, where *dσ* and *d*Φ represent the charge density and local potential, respectively. According to the formula *μ* *= e*Φ, the electrochemical potential *μ_F_* can move rigidly through the local potential Φ. On the electrode, the excess charge density can be expressed as the Equation (1) [54]:

(1) $$\Delta Q=\int_{-\infty }^{+\infty }D(E)[f(E)-f(E-e\Phi )]dE$$

in which *D*(*E*) represents the density of states (DOS), and *f*(*E*) represents the Fermi–Dirac distribution function. *E* is the relative energy of the Fermi level *E_F_*. According to the analytical Formula (1) of Δ*Q*, the quantum capacitance *C_Q_* can be calculated using Equation (2) [54,55]:

(2) $$C_{Q}=e^{2}\int_{-\infty }^{+\infty }D(E)F_{T}(E-e\Phi )dE$$

in which *F_T_*(*E*) represents the thermal broadening function, which can be described as the Equation (3):

(3) $$F_{T}(E)={\left(4k_{B}T\right)}^{-1}Sech^{2}(E/2k_{B}T)$$

*k_B_* is the Boltzmann constant, and the room temperature was set at 300 K.

The formation energies of single-vacancy germanene, double-vacancy germanene, and triple-B (N, P, S) atoms doped with single-vacancy germanene were calculated using the following Formulas (4)–(6):

(4) $$\Delta E_{SV}(x)=E_{SV-Ger}(x)-(n-1)\mu_{Ge}$$

(5) $$\Delta E_{DV}(x)=E_{DV-Ger}(x)-(n-2)\mu_{Ge}$$

(6) $$\Delta E_{3-B(N,P,S)}=E_{SV-Ger-3B(N,P,S)}-(n-3)\mu_{Ge}-3\mu_{B(N,P,S)}$$

Among them, *E_SV-Ger_*, *E_DV-Ger_*, *E_SV-Ger-3B_*_(*N*, *P*, *S*)_ represent the total energy of single/double-vacancy germanene, and single-vacancy germanene doped with triple-B (N, P, S) atoms, respectively. *μ_Ge_* and *μ_B_*_(*N*, *P*, *S*)_ are the energy of the single Ge atom in pristine germanene and B_12_ (N_2_, P_4_, S_8_) molecules in the gas phase, respectively, and n is the total number of atoms in pristine germanene.

The adsorption energy of the metal atoms (Ti, Au, Ag, Cu and Al) on pristine germanene was calculated by the following Formula (7):

(7) $$\Delta E_{ad}=E_{Ger-m}-n\mu_{Ge}-\mu_{m}$$

in which *E_Ger-m_* is the energy of metal atoms adsorbed on germanene and *μ_m_* is the energy of a single metal atom.

## 3. Results and Discussion

**Figure 1:**
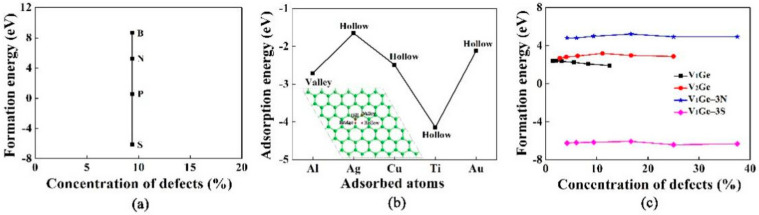
Formation energies of (**a**) triple-B (N, P, S) doped with single-vacancy germanene at a defect concentration of 9.4%; (**b**) Adsorption energies of the most stable configuration of metal atoms adsorbed on pristine germanene at a defect concentration of 3.1%, the results were obtained within the supercell 4 × 4; (**c**) Formation energies of single-vacancy, double-vacancy and triple-N (S) doped with single-vacancy germanene.

Figure 1a shows the formation energy of single-vacancy germanene doped with triple-B (N, P, S) atoms at a doping concentration of 9.4%. The formation energies of B, N, P and S doped with single-vacancy germanene were 8.684 eV, 5.256 eV, 0.575 eV and −6.149 eV, respectively. Triple-S atoms doped had the lowest formation energy, which indicates that it is a stable doping structure. The S atom could be used as an ideal doping atom for germanene-based electrode materials. In addition, N, P and S were better dopants than B. We could see that the dopants of N_2_, P_4_ and S_8_ as reference states were easier than the B-doping with B_12_.

Figure 1b shows the adsorption energy of Ti, Au, Ag, Cu and Al atoms at the most stable position of pristine germanene, with the adsorption concentration of 3.1%. In this study, the adsorption energies of the most stable adsorption sites for Ti, Au, Ag, Cu and Al were calculated to be −4.144 eV, −2.113 eV, −1.650 eV, −2.489 eV and −2.716 eV, respectively. The adsorption of germanene had four typical sites, which had stable or metastable binding with atoms, namely hill, valley, bridge and hollow sites in Figure 1b. It was found that the most stable positions of Ti, Au, Ag and Cu were hollow sites, however, the most stable position was the valley site for Al. According to the structural models, it could be concluded that the adsorbed atoms cause local deformation of the germanene layer. The Cu and Au atoms were totally embedded in the germanene and the Ge atoms in the next layer were pushed downward from the original positions. For instance, the distance between the adsorbed Cu atom and Ge atom in the next layer changed from 2.5 Å to 1.034 Å. Figure 1c shows the formation energy of single(double)-vacancy germanene, and single-vacancy germanene doped with triple-N and S atoms at different concentrations. The formation energy of single-vacancy was high because of the destruction of the Ge-Ge bond. For single-vacancy germanene, the formation energy increased and then decreased with the increase of defect concentration. The formation energies of double-vacancy germanene and single-vacancy germanene doped with triple-N or S atoms also had the same trend. The formation energy of triple-N doped with single-vacancy germanene was almost constant because the binding force between N and Ge atom was very strong and the energy was released, it had good stability.

**Figure 2:**
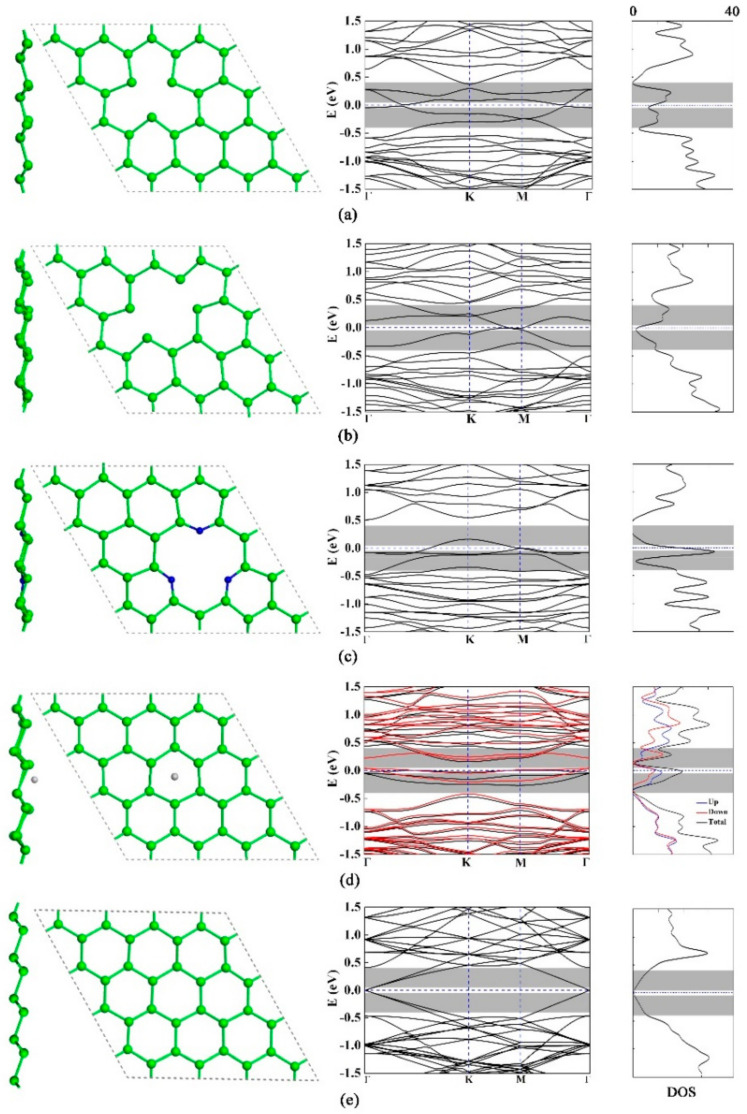
Atomic structures (left) of proposed germanene-based models, band structure (middle), and density of states (right) of the (**a**) single-vacancy, (**b**) double-vacancy, (**c**) triple-N doped with single-vacancy germanene, and (**d**) Ti atom adsorbed on pristine germanene with hollow site and (**e**) pristine germanene. The Fermi levels were indicated by the blue and horizontal dashed lines.

The electronic performance of defective germanene with the fully relaxed structure are shown in Figure 2. The significant characteristic of the six-fold or three-fold symmetric band structures could be expressed by the high symmetry lines of the Brillouin zone along Γ-K-M-Γ. The Figure shows the band structures and density of states of the four defect structures. As shown in Figure 2a,c, the Fermi level moved down because the band gaps were opened by the defects. Their band gaps were 0.08 eV and 0.39 eV, respectively. For the double-vacancy germanene in Figure 2b, the band gap near the Fermi level was zero. As shown in Figure 2d, the adsorption of Ti leaded to spin polarization. The symmetry of spin-up and spin-down was destroyed near the Fermi level, and the asymmetric density of states caused by Ti-3d orbit. The band gap at the Dirac point was open, and local defect states were formed in the band gap. It could be observed that the all the four defect structures introduced local states near the Fermi level, and the peaks corresponded to the partial flattening of the bands.

**Figure 3:**
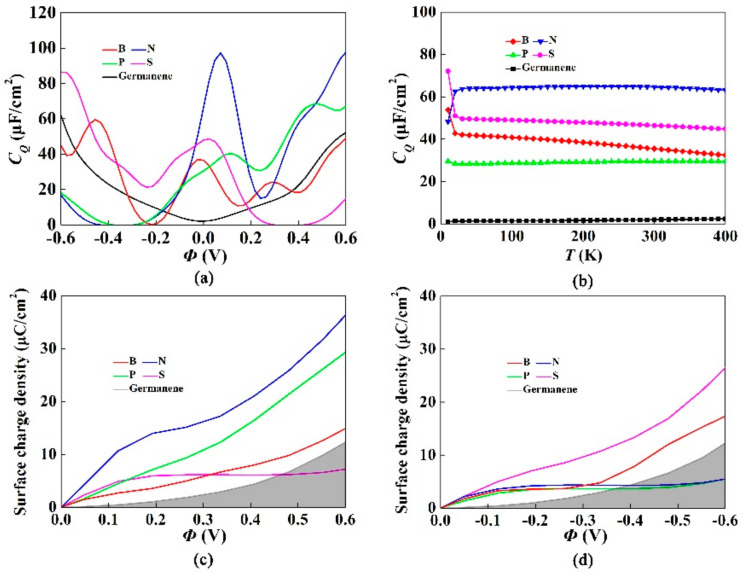
(**a**) Calculated quantum capacitance of the triple-B (N, P, S) doped with single-vacancy germanene; (**b**) Change of *C_Q_* with temperatures in the scope of 0 K–400 K, the consequences gained in 4 × 4 supercell with a doping concentration of 9.4%; (**c**,**d**) Surface charge density vs. Φ between −0.6 V and 0.6 V.

Figure 3a shows the effect of triple-B (N, P, S) doped with single-vacancy germanene on the quantum capacitance. The quantum capacitance of pristine germanene was approximately zero when the local potential (Φ) was 0 V. Moreover, it had the same trend on both sides of the Fermi level. It was due to the linear dispersion near the Dirac point. For single-vacancy germanene doped with B, N, P and S atoms, the local maximum values of quantum capacitance were 37.0 μF/cm^2^ (−0.024 V), 97.2 μF/cm^2^ (0.072 V), 40.3 μF/cm^2^ (0.12 V) and 48.6 μF/cm^2^ (0.024 V), respectively. Therefore, the quantum capacitance of germanene was significantly improved by the dopants. It was ascribed to the localized state produced by dopants and single vacancy near the Dirac point. To understand the local state near the Fermi energy of the doped atoms, we studied the effect of temperature on *C_Q_* in Figure 3b. We set the temperature range from 0 K to 400 K and calculated the *C_Q_* with zero bias. The quantum capacitance of pristine germanene remained almost unchanged, close to 1.97 µF/cm^2^. The quantum capacitance of single-vacancy germanene doped with B, N, P and S atoms also did not change significantly with temperature. The weak change of *C_Q_* with temperature was connected to the density of states near the Fermi level. According to the calculated charge accumulation effect in Figure 3c,d, the charge accumulation of single-vacancy germanene doped with N, P greatly increased under the positive potential. Both B and S had an obvious enhancement effect under the negative potential.

**Figure 4:**
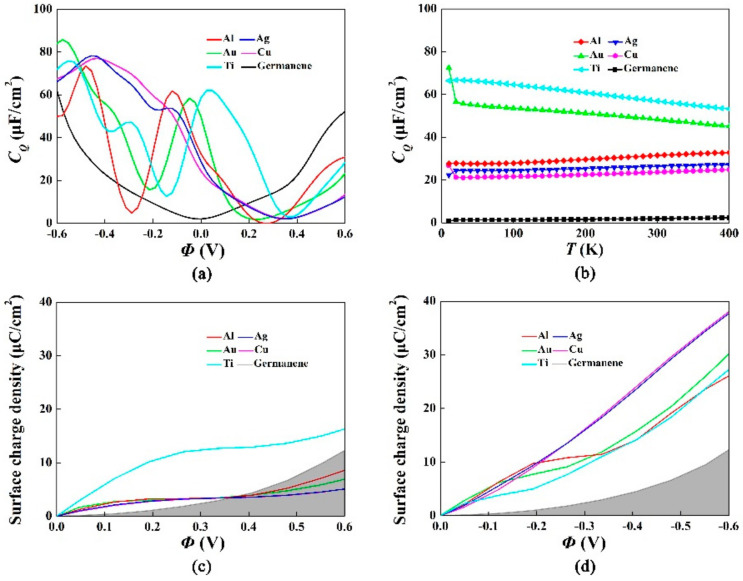
(**a**) Quantum capacitance of the pristine germanene adsorbed Ti, Au, Ag, Cu, Al at the most stable configuration; (**b**) Change of *C_Q_* with temperature in the scope of 0 K–400 K, the consequences gained in 4 × 4 supercell at a doping concentration of 9.4%; (**c**,**d**) Surface charge density vs. Φ between −0.6 V and 0.6 V.

Figure 4a shows the calculated quantum capacitance of the pristine germanene adsorbed metal atoms at the most stable configuration. For the adsorption of Ti, Au, Ag, Cu and Al on pristine germanene, the local maximum values of the *C_Q_* near 0 V were 62.0 μF/cm^2^ (0.024 V), 58.4 μF/cm^2^ (−0.048 V), 53.7 μF/cm^2^ (−0.144 V), 77.1 μF/cm^2^ (−0.432 V), and 61.8 μF/cm^2^ (−0.120 V), respectively. The quantum capacitance of the germanene observably increased with adsorption of metal atoms. Among these metal atoms, the adsorption effects of Ti, Au and Al were better than Ag and Cu.

Figure 4b shows the relationship between the quantum capacitance and temperature. The quantum capacitance of pristine germanene adsorbed metal atoms did not change significantly with temperature. Figure 4c,d show the surface charge density versus the potential drop of pristine germanene adsorbed different metal atoms, with a potential range of −0.6–0.6 V. The charge accumulation was obvious under the positive potential for Ti and the negative potential for Ag and Cu. The *C_Q_* of metal-adsorbed germanene changed with the increase of electrode potential, which indicated that the DOS also varied with the potential. According to the formula 1/*C_T_* = 1/*C_D_* + 1/*C_Q_*, the total interface capacitance changed with the quantum capacitance. Pristine germanene had a low quantum capacitance at a small potential, which significantly affected the total interface capacitance. The improvement of quantum capacitance was obtained by the adsorption of metal atoms, and it significantly improved the total interface capacitance.

**Figure 5:**
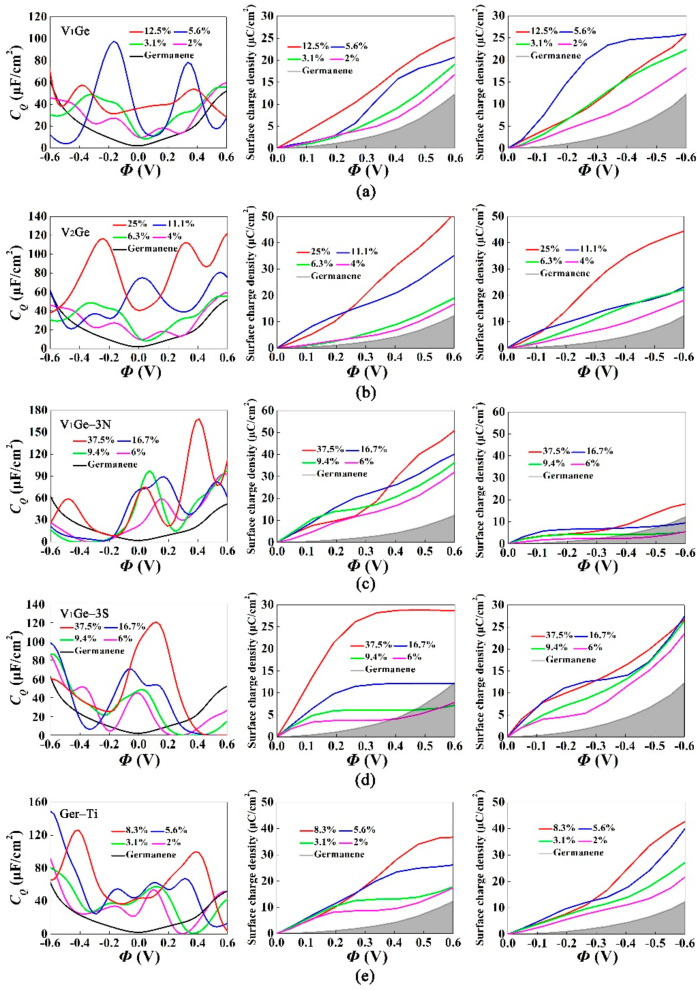
Calculated quantum capacitance (left) and surface charge density vs. potential drop (−0.6 V~0.6 V) with different concentrations of (**a**) single-vacancy, (**b**) double-vacancy, (**c**) triple-N doped with single-vacancy germanene, (**d**) triple-S doped with single-vacancy germanene and (**e**) pristine germanene adsorbed Ti at the most stable configuration.

Figure 5 shows the quantum capacitance and the surface charge density versus the potential drop for the five defect structures with different concentrations, at the potential range of −0.6–0.6 V. In Figure 5a, when the single-vacancy concentration was 5.6%, the quantum capacitance was 97.6 μF/cm^2^ at −0.168 V. The charge accumulation effect was obviously enhanced under the positive and negative bias. The phenomenon was caused by the increase of effective states near the Fermi level. As the concentration of double-vacancy germanene raised from 4% to 25% in Figure 5b, the local peak value of the quantum capacitance raised from 27.4 μF/cm^2^ (−0.168 V) to 116.6 μF/cm^2^ (−0.240 V). The charge accumulation increased under the positive and negative potential. It was observed that the disappearance of dangling bonds around the vacancy means that the localized states were introduced by double-vacancy at the low concentration near the Fermi level.

Figure 5c,d show the *C_Q_* and surface charge density of single-vacancy germanene doped with triple-N and S atoms. When the concentration raised from 6% to 37.5%, the peak value of the quantum capacitance increased from 58.7 μF/cm^2^ (0.144 V) to 168.3 μF/cm^2^ (0.408 V) for triple-N doping and increased from 45.1 μF/cm^2^ (0 V) to 120.5 μF/cm^2^ (0.120 V) for triple-S doping. The increase of the local states leaded to the enhanced quantum capacitance at a small voltage. N-doping had the best charge accumulation effect under the positive potential, while S-doping had it under the negative potential.

As shown in Figure 5e, when the concentration raised from 2% to 8.3%, the maximum value of the quantum capacitance changed from 53.9 μF/cm^2^ (0.096 V) to 125.9 μF/cm^2^ (−0.408 V). In contrast to pristine germanene, the low concentration of Ti-doping didn’t significantly affect the electronic structure of germanene. As the Ti concentration increased, these defective states widened above the Fermi level owing to the coupling of the defective states, thus resulting in an increase of the quantum capacitance. As the Ti concentration increased, the charge accumulation effect improved irrespective of positive and negative potential. This was because of the formation of a peak in the density of states at low concentrations near the Fermi level.

**Figure 6:**
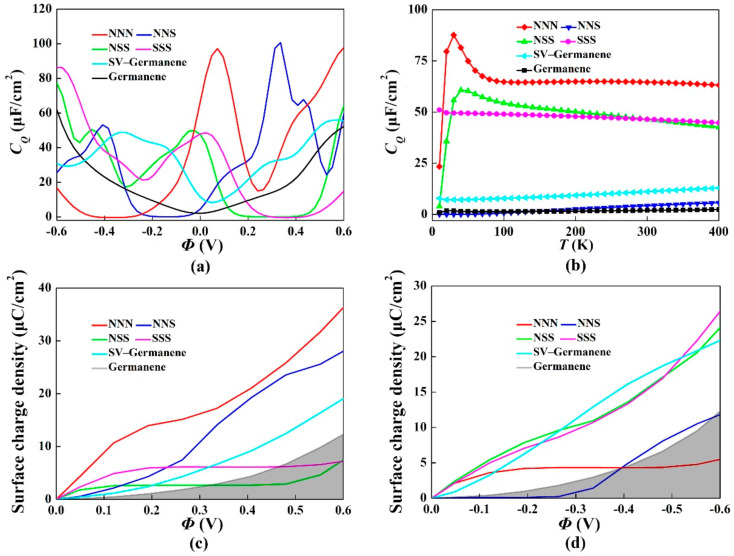
(**a**) Calculated quantum capacitance of the N/S co-doped with single-vacancy germanene; (**b**) Change of *C_Q_* with temperatures in the scope of 0 K–400 K, the consequences gained in 4 × 4 supercell; (**c**,**d**) Surface charge density vs. Φ between −0.6 V and 0.6 V.

For the N/S co-doping system with different ratios, including 3:0 (NNN-doping), 2:1 (NNS co-doping), 1:2 (NSS co-doping), and 0:3 (SSS-doping), the *C_Q_* and surface charge density vs. Φ were shown in Figure 6. It was apparent that all the *C_Q_* values were better than that of pristine germanene in Figure 6a. With the variation of N/S ratios, the peak of *C_Q_* raised from 48.6 μF/cm^2^ (0.024 V) to 49.8 μF/cm^2^ (−0.024 V) for the NSS co-doping, 100.7 μF/cm^2^ (0.336 V) for the NNS co-doping and to 97.2 μF/cm^2^ (0.072 V) for the NNN-doping. Figure 6b shows the relationship between quantum capacitance and temperature. The quantum capacitance of N/S co-doped with single-vacancy germanene didn’t change significantly with temperature. The surface charge density was clearly better than that of pristine germanene in Figure 6c,d. For the NNN-doping, the surface charge density increased under the positive potential. The trend of surface charge density of NSS co-doping was similar to that of SSS-doping, and they showed a monotonous increasing trend under the negative potential.

**Figure 7:**
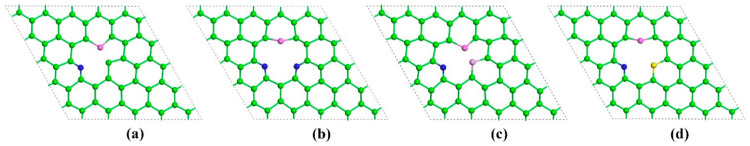
The atomic structures of proposed germanene-based models including single-vacancy germanene co-doped with the (**a**) NAl, (**b**) NNAl, (**c**) NPAl and (**d**) NSAl.

**Figure 8:**
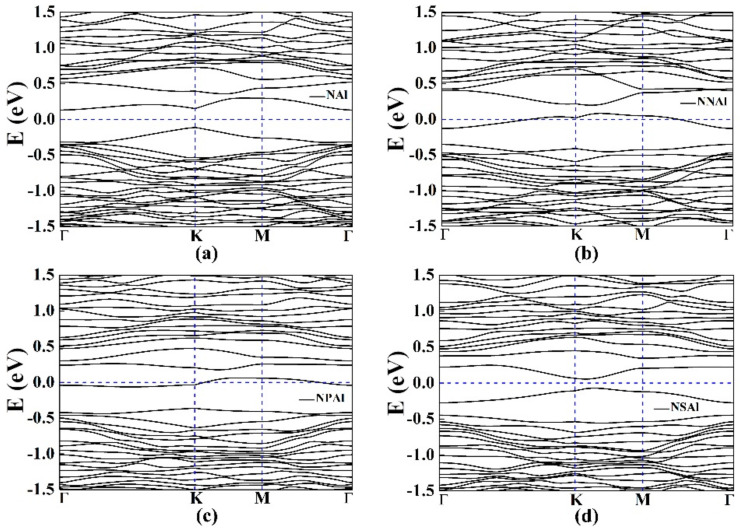
The band structures of single-vacancy germanene co-doped with the (**a**) NAl, (**b**) NNAl, (**c**) NPAl and (**d**) NSAl.

**Figure 9:**
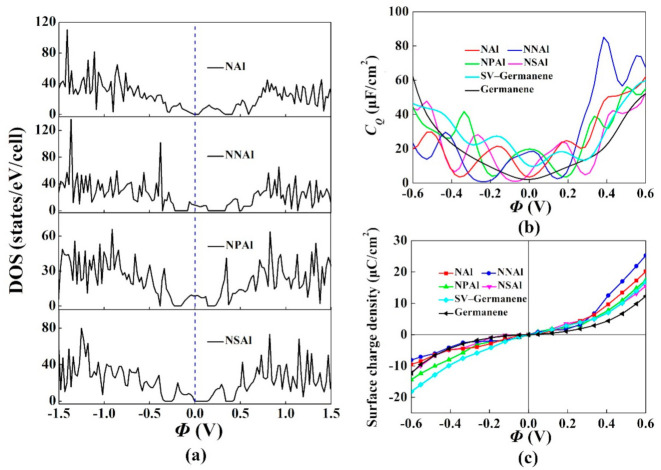
(**a**) Density of states (DOS) of single-vacancy germanene co-doped with N/P/S/Al; (**b**) Calculated quantum capacitance of the N/P/S/Al co-doped with single-vacancy germanene; (**c**) Surface charge density vs. Φ between −0.6 V and 0.6 V.

We also explored the effect of NAl, NNAl, NPAl and NSAl co-doped with single-vacancy germanene on *C_Q_*. They all introduced localized states near the Fermi level. N was one of the most common elements to regulate the physicochemical properties of germanene. With the introduction of N atoms, the hybrid states of Al atom tended to be consistent. Moreover, some studies had shown that replacing the atoms around the metal with N atoms could further stabilize the metal atoms at the defect site, thereby exhibiting higher chemical activity [56]. The optimized structure models, the corresponding band structures and the total DOS were shown in Figure 7, Figure 8 and Figure 9. For NNAl and NPAl co-doping, the bandgaps at the Dirac point were opened, the bandgaps were 0.120 eV and 0.124 eV, respectively. The Fermi level shifted down to the valence zone. The results show that NNAl and NPAl co-doping were more conductive than NAl and NSAl co-doping, which was consistent with the DOS in Figure 9. The quantum capacitance of single-vacancy germanene co-doped with N/P/S/Al atoms was shown in Figure 9b. For the NAl, NNAl, NPAl and NSAl co-doping, the local maximum values of the *C_Q_* were 21.4 μF/cm^2^ (−0.168 V), 18.5 μF/cm^2^ (0.024 V), 19.8 μF/cm^2^ (0 V), 24.0 μF/cm^2^ (0.168 V), respectively. Evidently, the high quantum capacitance was attributed to the localized states near the Fermi level [57]. This demonstrated that the co-doped with single vacancy could ameliorate the *C_Q_* of pristine germanene. Figure 9c represents the surface charge density versus Φ. The charge accumulation exhibited an enormous improvement for NNAl co-doping with the positive potential.

**Figure 10:**
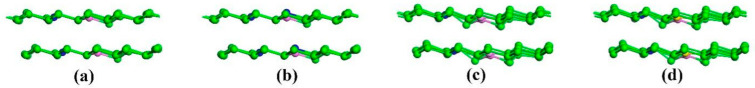
The side views for atomic multilayered structures of proposed germanene-based models including single-vacancy germanene co-doped with the (**a**) NAl, (**b**) NNAl, (**c**) NPAl and (**d**) NSAl.

**Figure 11:**
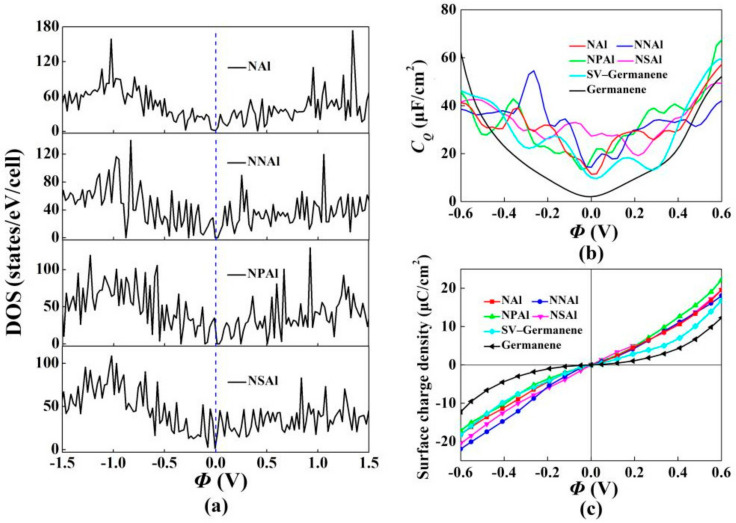
(**a**) Density of states (DOS) of multilayered structures of single-vacancy germanene co-doped with N/P/S/Al; (**b**) Calculated quantum capacitance of multilayered structures of the N/P/S/Al co-doped with single-vacancy germanene; (**c**) Surface charge density with Φ between −0.6 V and 0.6 V.

The majority of theoretical and experimental studies had focused on the monolayer germanene due to its simplicity. However, the understanding of the modified properties due to interaction between layers occurred in multilayer structures was also important in supercapacitor electrode application [58]. This work researched structures, stability, electronic properties and *C_Q_* of multilayer germanene to clearly understand the properties of modified germanene under the interaction with germanene layers. Therefore, we modified the multilayered structures of NAl, NNAl, NPAl and NSAl co-doped with germanene. The optimized structures were shown in Figure 10. The *C_Q_* and surface storage charge of the pristine/single-vacancy and four kinds of multilayer germanene were contained in Figure 11b,c. For quantum capacitance of multilayer structures co-doped with NAl, NNAl, NPAl and NSAl system, the local maximum values were 39.1 μF/cm^2^ (−0.336 V), 54.6 μF/cm^2^ (−0.260 V), 42.8 μF/cm^2^ (−0.360 V) and 33.0 μF/cm^2^ (−0.072 V), respectively. Due to the interaction between dopants of adjacent layers, the *C_Q_* of multilayer was better than that of monolayer structures. The charge accumulation was enhanced at the positive potential for NPAl co-doping, and NNAl co-doping had the best charge accumulation effect under the negative potential.

## 4. Conclusions

The effects of doping/co-doping, vacancy defects and multilayered structures on the electronic structure and quantum capacitance of germanene were studied by first-principles calculations. The adsorption of Ti, Au, Ag, Cu and Al on pristine germanene were also examined.

The results show that the doping of B, N, P and S atoms and the adsorption of metal atoms significantly improved the quantum capacitance of germanene. It was found that these ways remarkably alter the band by introducing localized states and/or Fermi level shifts near the Dirac point. Analysis of the doping effect of triple-B (N, P, S) with single-vacancy germanene on the quantum capacitance shows that triple-N doping could significantly improve the quantum capacitance of germanene. Secondly, the structures of pristine germanene adsorbed metal atoms were modified, the calculated *C_Q_* values suggested that the adsorbed Ti and Al atoms systems had the best performance. Thirdly, we also found that as the defect concentration increased, the quantum capacitance showed a monotonic increase. Both NNN-doping and NNS co-doping had obvious effect on the improvement of quantum capacitance. Finally, we modified the monolayer and multilayer structures of germanene co-doped with NAl, NNAl, NPAl and NSAl. The results indicated that the interaction between layers increased the quantum capacitance. Therefore, vacancies and doping play a momentous part in improving the quantum capacitance of germanene.

## Data Availability

The data presented in this study are available on request from the corresponding author.
